# Evolution analysis of heterogeneous non-small cell lung carcinoma by ultra-deep sequencing of the mitochondrial genome

**DOI:** 10.1038/s41598-017-11345-3

**Published:** 2017-09-11

**Authors:** Wafa Amer, Csaba Toth, Erik Vassella, Jeannine Meinrath, Ulrike Koitzsch, Anne Arens, Jia Huang, Hannah Eischeid, Alexander Adam, Reinhard Buettner, Andreas Scheel, Stephan C. Schaefer, Margarete Odenthal

**Affiliations:** 1 Institute of Pathology, University Hospital of Cologne, Cologne, Germany; 2 Institute of Pathology, University Hospital of Bern, Bern, Switzerland; 3 Qiagen Inc, Hilden, Germany; 4 Center of Integrative Oncology, University Clinic of Cologne and Bonn, Cologne, Germany; 5 Lung Cancer Group Cologne, University Hospital of Cologne, Cologne, Germany; 6 Center of Molecular Medicine of Cologne, University of Cologne, Cologne, Germany

## Abstract

Accurate assessment of tumour heterogeneity is an important issue that influences prognosis and therapeutic decision in molecular pathology. Due to the shortage of protective histones and a limited DNA repair capacity, the mitochondrial (mt)-genome undergoes high variability during tumour development. Therefore, screening of mt-genome represents a useful molecular tool for assessing precise cell lineages and tracking tumour history. Here, we describe a highly specific and robust multiplex PCR-based ultra-deep sequencing technology for analysis of the whole mt-genome (wmt-seq) on low quality-DNA from formalin-fixed paraffin-embedded tissues. As a proof of concept, we applied the wmt-seq technology to characterize the clonal relationship of non-small cell lung cancer (NSCLC) specimens with multiple lesions (N = 43) that show either different histological subtypes (group I) or pulmonary adenosquamous carcinoma as striking examples of a mixed-histology tumour (group II). The application of wmt-seq demonstrated that most samples bear common mt-mutations in each lesion of an individual patient, indicating a single cell progeny and clonal relationship. Hereby we show the monoclonal origin of histologically heterogeneous NSCLC and demonstrate the evolutionary relation of NSCLC cases carrying heteroplasmic mt-variants.

## Introduction

Tumour tracking and evolution analysis to identify the intra-tumour clonal structure or history of multiple tumour lesions within the same patient are currently evolving into important diagnostic tools for the precision treatment of malignant neoplasias^1–3^.

The human mitochondrial genome is a circular DNA molecule that encompasses ~16.5 kbp and contains 37 genes [http://www.mitomap.org]. Each mitochondrion contains 10–15 copies of mitochondrial (mt) DNA, which is predisposed to a 10-fold higher accumulation of mutations than nuclear DNA^4^. This is due to the fact that the mitochondria are exposed to high levels of reactive oxygen species (ROS)^5, 6^. Furthermore, mitochondria lack protective histones and an efficient DNA repair system, which results in limited defence mechanisms against endogenous or exogenous damaging agents such as oxidative stress and leads to a high mutation rate^7, 8^. During tumour development, some mutated mtDNA copies may confer selective advantage or disadvantage on tumour cells during mt-DNA replication, cell growth and infiltration, resulting in clonal expansion or loss of the mutated mtDNA copies. Therefore, mutations do not affect only a proportion of the mitochondrial genome copies (heteroplasmic mutations), but often affect all copies of a tumour lesion (homoplasmic mutations)^9, 10^. The resulting high mutation rate and mt-variability, the high number of mt-DNA copies within each cell, and the fact that most of the somatic mtDNA mutations are homoplasmic^9, 10^, make the mt-genome an ideal target for tumour cell tracking^11, 12^.

In the present study, we established an ultra-deep sequencing approach, identifying mt-variants of the entire mt-genome on formalin-fixed paraffin-embedded (FFPE) tumour lesions. This novel technology of ‘whole mitochondrial DNA ultra-deep sequencing’ (wmt-seq) was applied to non-small cell lung cancer (NSCLC), which represents 80% of all lung cancer. The two main histological subtypes of NSCLC are adenocarcinoma (AD), which accounts for 50% NSCLC, and squamous cell carcinoma (SQ), which accounts for 40%^13^. However, NSCLC exhibits a variety of morphological and molecular features^14^. Moreover, tumour-heterogeneity is a common and well-recognised phenomenon in NSCLC, much more than in other solid tumours^15^. The accurate clonal assessment of NSCLC to either distinguish clinically challenging synchronous and metachronous tumours or differentiate between multiple primary tumour lesions from metastases is an essential basis for prognostic estimation and therapeutic decision^16^.

In order to study the clonal relationship, we used two NSCLC cohorts characterized by i) multiple lesions with different histology within the same patients or ii) tumours with heterogeneous, adenosquamous differentiation. Hereby we clearly show that an ultra-deep sequencing technology of the entire mt-genome (wmt-seq) is a suitable molecular tool for tumour history tracking on pathologically processed FFPE material.

## Results and Discussion

In order to track tumourigenesis on FFPE archived material, we developed a novel approach for comprehensive mtDNA mutation analysis using a multiplex PCR-based ultra-deep sequencing approach on multifocal NSCLC lesions of different histological growth patterns.

**Figure 1:**
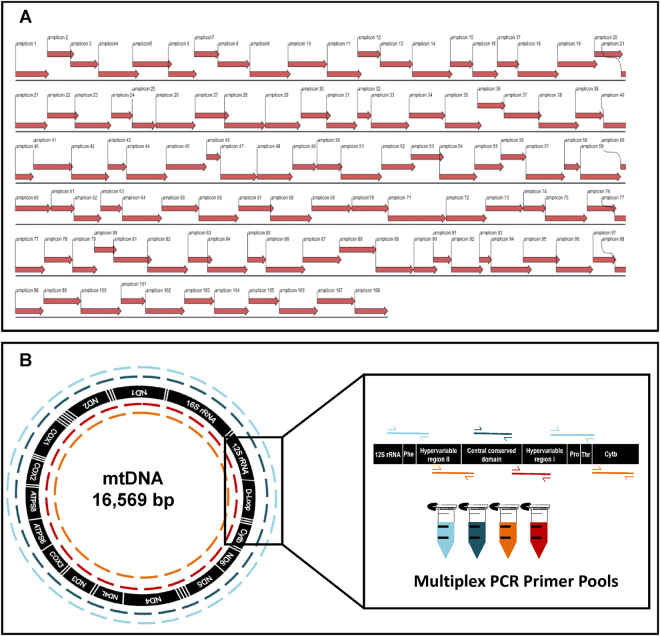
Scheme of primer design and the multiplex PCR-based approach to mt-genome enrichment. Primer sets (Table S2) were designed (**A**) generating 108 amplicons spanning the whole mitochondrial genome (**B**). Four primer pools, each including 27 primer sets, were applied to multiplex PCR according to the GeneRead multiplex PCR design of Qiagen, to ensure that overlapping amplicons were generated in separate reaction mixes.

For molecular tumour tracking, a PCR-based NGS of the entire mt-genome was established. To generate amplicons of a low size (around 60-200 bp), 108 primer sets, spanning the entire mtDNA (Fig. 1A) were designed according to the mt-sequence of accession no. NC_012920 or taken from previously published primer sets (Supplemental Table S1). Primers were designed to generate overlapping amplicons of 160 bp average length to guarantee robust multiplex PCR (Fig. 1B). This design allowed an efficient amplification of small quantities of highly fragmented DNA (Supplemental Figure S1) as extracted from FFPE material^17^. Though formalin causes high nucleic acid fragmentation and nucleotide modification such as cytosine amination that leads to a C > T/ G > A exchange during PCR reactions, it is used world-wide in diagnostic routine-processing of tissues. Therefore, our approach is of particular interest for analysis of clinical, formalin-fixed samples. Circulating DNA, isolated from plasma and serum samples, is also of low quantity and quality^18, 19^. Since in the recent past circulating mtDNA has been highlighted as a prognostic tool in cancer diagnostics^20, 21^, our technical approach benefits from the high efficiency of whole mt-DNA sequencing (wmt-seq) on short DNA fragments.

Previous NGS-based approaches to mtDNA analysis used long-range PCR amplicons, and worked only on native material^22, 23^. Others focused on the analysis of the mt-control region (D-LOOP), which encompasses a hotspot region but only covers 7% of the total mt-genome^11, 12^. The presented robust multiplex PCR, however, was used for PCR-based target enrichment, providing an approach to wmt-seq that can be applied to different sources of DNA.

### Application of ultra-deep wmt-seq on NSCLC lesions

Forty-three nodules from 19 patients with NSCLC were studied for tumour heterogeneity by wmt-seq. The FASTQ files generated by wmt-seq of 43 samples were applied to bioinformatics analysis. The sequences were mapped against the entire human reference genome hg19 to exclude the possibility that nuclear pseudogenes, which have a high homology to parts of the mt-genome, were recognised. As a threshold for variant calling, a minimum read depth was set to 30 and the minimum variant frequency was set to 5%. Polymorphisms were recognised using the MITOMAP (www.mitomap.org), dbSNP-v138 (www.ncbi.nlm.nih.gov/SNP), and HAPMAP_phase_3 (hapmap.ncbi.nlm.nih.gov) databases.

The data analysis output generated an average of 484 × 10^3^ reads per sample. A total of 473 × 10^3^ reads were mapped to the mtDNA reference sequence, showing that 96% of the mt-genome was covered by a mean read depth of 3.000 reads per 100 bp (Supplemental Table S2). Furthermore, the high sequencing performance was demonstrated by nearly 98% run specificity, proven by only 2.5% off-t﻿argets read﻿s ﻿(Supplemental Table S3).

The good coverage of the mt-genome and the low rate of site-off reads demonstrated the efficiency of the designed primer sets and a good run performance for the analysis of the entire mt-genome.

### mt-variants identified in NSCLC by wmt-seq

**Figure 2:**
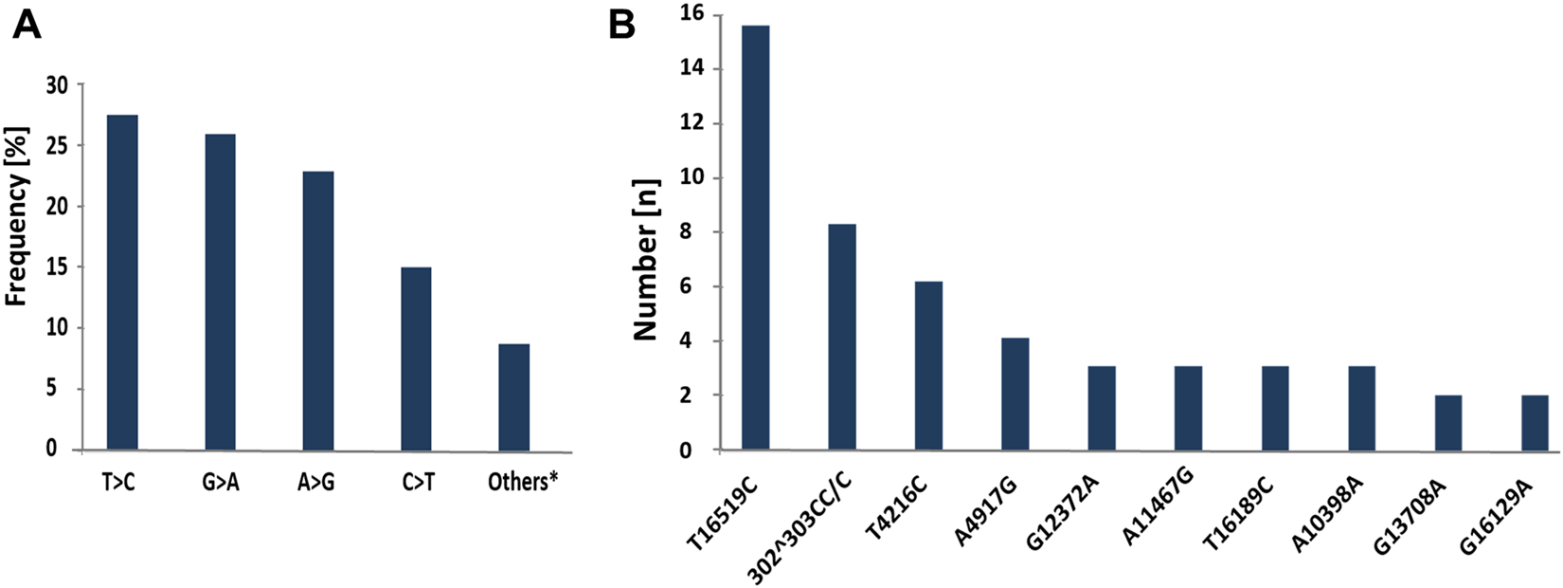
Disease-related and base pair transition mt-mutations. Overall frequency of base pair transitions, found in NSCLC. T > C:G > A are the most common base pair transitions. Other*: Indels and transversion mutations (**A**). Homoplasmic polymorphisms, previously shown to have a functional impact in different cancer types(MITOMAP database) were frequently detected in the NSCLC cohort (n = number of positive samples) (**B**).

After data filtering, a total of 640 variants were identified (Supplemental Figure S2). Most of these mutations were T > C/A > G, G > A/C > T base transitions (Fig. 2A) as shown by Kennedy *et al*.^24^. In agreement with previous reports^9, 10, 25^, the highest frequency of variants was observed in the D-LOOP regulatory site, which is responsible for the replication and expression of mtDNA (178/640, 28%). Furthermore, a high frequency of variants was found in the genes encoding for the respiratory chain complexes (Supplemental Figure S2). These mutations could lead to an abnormal metabolism as well as to altered apoptosis^26, 27^. The defined mutations are listed in Supplemental Table S4.

The majority of detected variants were homoplasmic (520/640; 81%) or highly heteroplasmic, as reported in previous studies^9, 25^. Most of the mt-variants might have occurred before tumourigenesis, because germline mtDNA polymorphisms generally reach a relatively high percentage of heteroplasmy or even homoplasmy during cellular phenotype development^28^. Some of these mtDNA polymorphisms (Fig. 2B) were previously shown to be disease-related and should be considered as possibly pathogenic (c.f. MITOMAP database)^29, 30^. Furthermore, in agreement with the studies of Brandon *et al*. our data revealed that most of the identified tumour associated mtDNA variants (Fig. 2B) are frequent in the general population^28^. Notably, two of the detected mtDNA population polymorphisms, namely mtDNA mutations at nucleotides 10398 and 16189 (Fig. 2B), have been shown to be associated with an increased risk of both breast^31^ and endometrial cancer^32^. The heteroplasmic T > C exchange at position 16189 is located in a hypermutable polyC stretch (16184^16193). This mt-microsatellite region as well as the one at position 302^315 are therefore appropriate regions to detect mt-DNA instability, which was shown previously to be associated with tumour malignancy^33, 34^.

Functional mtDNA polymorphisms may help tumours to adapt to new environments and actively grow in metastatic oxygen-rich conditions^28, 35^. Therefore, these mtDNA polymorphisms may become fixed and shift from initially heteroplasmic to homoplasmic mutations. Somatic tumour-specific mtDNA mutations inhibit oxidative phosphorylation, increase ROS production, and promote tumour cell proliferation. These somatic mutations may be lost during subsequent tumour oxygenation by replicative segregation, with the cell turning back towards the more oxidative mtDNA genotype favoured in the metastatic environment^28, 35^.

### NSCLC tumour-tracking and clonality analysis by wmt-seq

Tracking the tumour cell lineage was not possible on samples that carried only homoplasmic mtDNA polymorphisms (i.e. #02, #04, #10 and #11). However, sample sets that harboured heteroplasmic somatic mutations, occurring in all of the lesion’s individual nodules (trunk mutations), in a subset (branch mutations), or only in one individual nodule (private mutations) could be considered for evolution analysis.

**Figure 3:**
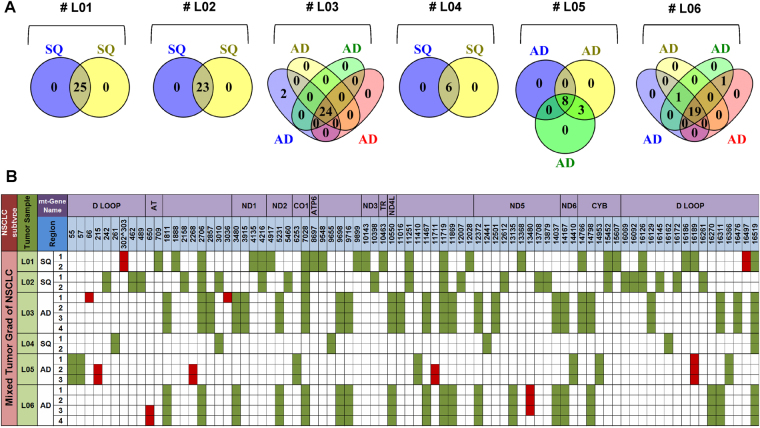
Global annotated mt-variants in tumour lesions with different growth patterns (group I). Common mt-variants (intersection area), adaptive mt-polymorphisms and somatic mutants occurring in NSCLC tumour lesions of cases #01–#06 are illustrated in Venn diagrams (**A**). Variants are shown according to their mt-genomic location and gene regions (**B**). Adaptive mt-polymorphisms common in all lesions of individual sample are labelled in green, whereas somatic mt-mutations with pathogenic impact on some lesions are shown in red (c.f. Supplemental Data and Table S4).

**Figure 4:**
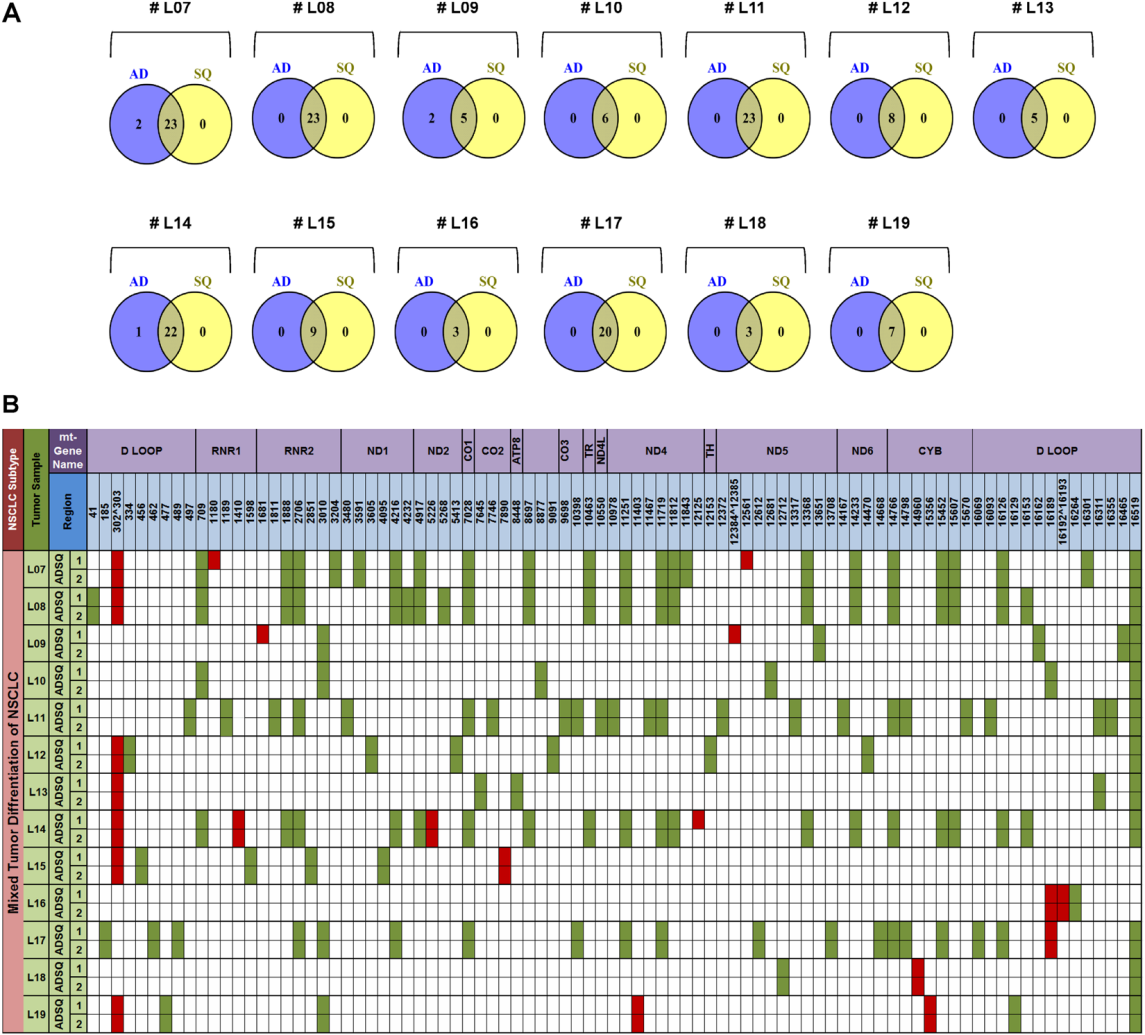
Global annotated mt-variants in mixed ADSQ samples (group II). Common mt-variants (intersection area), adaptive mt-polymorphisms and somatic mutants occurring in NSCLC tumour lesions of cases #07–#19 are illustrated in Venn diagrams (**A**). Variants are shown according to their mt-genomic location and gene regions (**B**). Adaptive mt-polymorphisms common in all lesions of individual sample are labelled in green, whereas somatic mt-mutations with pathogenic impact on some lesions are shown in red (c.f. Supplemental Data and Table S4).

Private mutations were only detected in single tumour nodules of particular cases (e.g.66 del-G and G3036A mutations in sample #03, T1180C and G12561A mutations in sample #07, G1681A and 12384^12385-ins C mutations in sample #09, and G12125A mutation in sample #14) (Figs 3 and 4, Supplemental Table S4). Most samples (i.e. #01, #05, #07, #08, #12, #13, #14, #15, #16, #17, #18 and #19), showed trunk mutations in the different nodules, proving a common cell of origin of the individual nodules (Figs 3 and 4, Supplemental Table S4). Moreover, branch mutations were detected in tumour nodules of sample #06, TN3 and TN4, which harboured a T650C mutation, whereas TN1, TN2 and TN3 carried a G13480A mutation (Supplemental Table 25, Supplemental Figure S3).

Notably, branch mutations in tumour nodules TN2 and TN3 of sample #05 at positions A215G, G2268A and G11711A, suggesting that TN2 and TN3 are subclones, derive from TN1. The increasing frequencies of the accumulating additional mutations show that they became dominant over time and provide insights about the evolutionary mechanisms that drive neoplastic progression (Supplemental Table S4, Supplemental Figure S3).

Thus, heteroplasmic somatic mutations indicate a clonal expansion of mtDNA mutants, which become either intra- and intercellularly dominant or submissive (Supplemental Table S4). This is in concordance with our findings that the adenocarcinoma and squamous carcinoma components of the adenosquamous NSCLC sample set (ADSQ-NSCLC, group II, Table 1) also share identical genomic hot-spot mutations e.g. in the TP53 and EGFR genes^36^.

**Figure 5:**
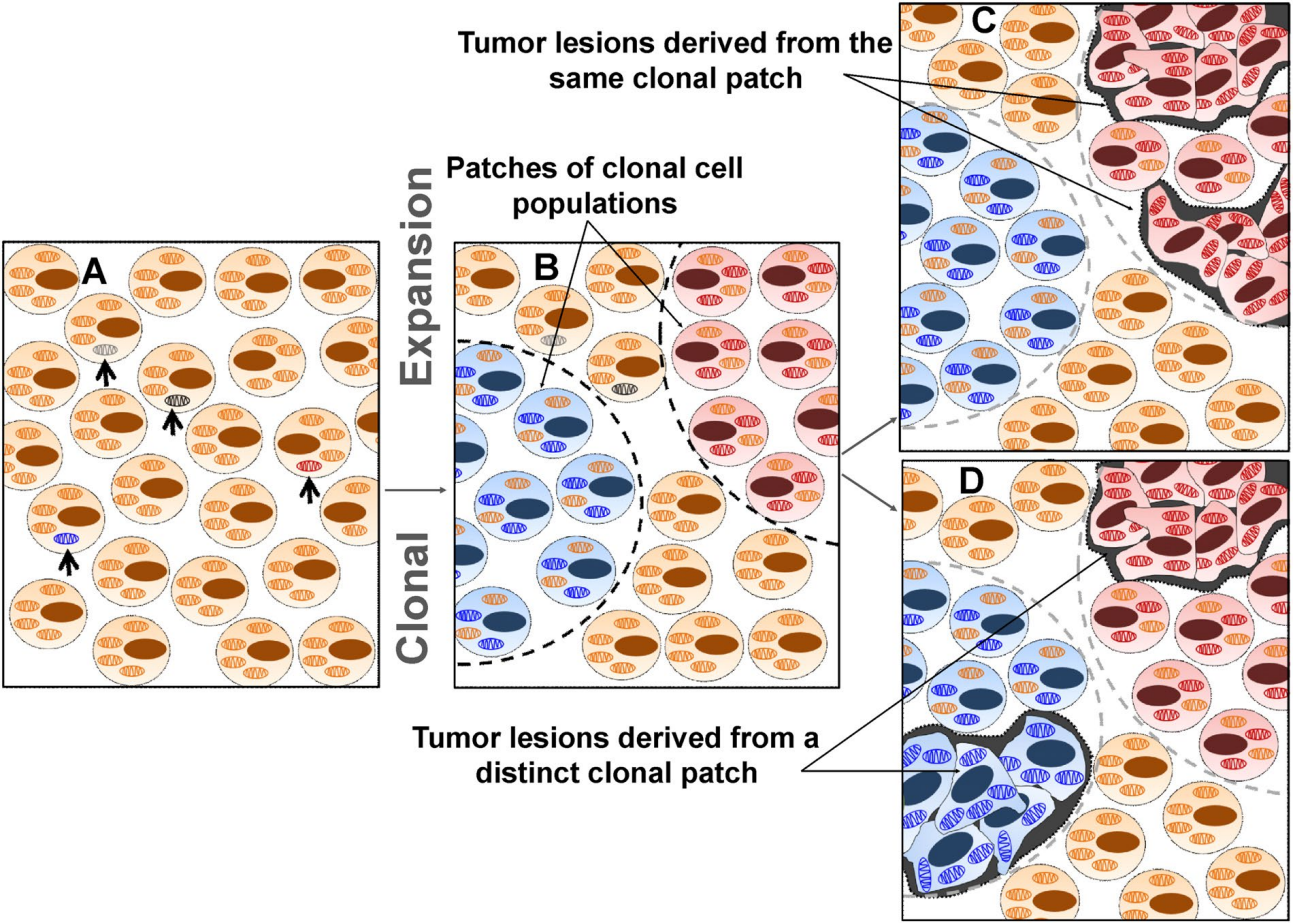
Clonal mt-mutation expansion of NSCLC tumour development. A stochastic mt-mutation (open arrows) arises at the germline or somatic level (**A**) and confers a cellular growth advantage likely to become dominant, leading to clonal patch formation (dashed boundaries) (**B**). Monoclonal tumour nodules, arising from a single patch, carry an identical mt-mutation pattern of homoplasmic and heteroplasmic mutations (indicated by the red mitochondria) (**C**). Tumour nodules, which arise from different clonal patches, contain distinct patterns of homoplasmic and heteroplasmic mt-mutations (**D**).

The mtDNA constantly predisposed to mutations that may either expand or be lost during tumour progression^9, 10, 38, 39^. As such, mt-mutations are discussed to acquire a selective replicative advantage during cellular development and become dominant, evolving a clonal cell population with homoplasmic mutants^9, 10, 37^. As previously shown, clonal homoplasmic expansion develops by crypt fission, forming large tumour patches with identical mt-mutations^38, 39^. In agreement, our data demonstrate that tumour nodules of the cases #01, #05, #07, #L08 and #12-19 arise within the clonal patch harbouring identical mt-mutations (Supplemental Table S4, Fig. 5).

In conclusion, the established multiplex PCR-based ultra-deep sequencing method may be considered as a novel molecular tool for the comprehensive analysis of the entire mt-genome. Though a limited number of private and branch variants, which we identified in different nodules, did not allow us to describe the complete evolutionary dynamics of tumor clonal networks^2, 3^, this technology provides for the first time a highly specific and sensitive approach to study the clonal relationship and tumour history on FFPE material of the pathology routine processing. This is of particular importance in terms of an appropriate tumour-specific treatment strategy when *de-novo* primary tumours and recurrent cancers have to be differentiated^12^.

Importantly, due to the tolerance of low DNA quantity and quality, the multiplex PCR-based wmt-seq technology might also be applicable to cell-free DNA approaches as a novel option to detect mitochondrial DNA alterations in various body fluids and to monitor cancer progression and mitochondrial disorders.

## Materials and Methods

### NSCLC biopsies and DNA extraction

A total of 43 FFPE NSCLC tumour lesions archived from 19 patients were included in this study (Table 1). Biopsies collected by the Institutes of Pathology at the University Hospital of Cologne, Germany, and the University Hospital of Bern, Switzerland, were used with the informed consent of the patients and in agreement with the local ethical guidelines as approved by the local ethical commissions (*‘Biomasota’ biomaterials collection, Az 13-091 and KEK Nr. 200/2014*). Multiple lung cancer lesions with different tumour grades or histology (group I) and tumours with intratumoural heterogeneity (group II) were characterized by immunohistology (Supplemental Figure S4).

**Table 1:** Histological characteristics of the 43 NSCLC lesions studied by mtDNA analysis.

NSCLC subtypes of different growth patterns (group I)				NSCLC of ADSQ subtype (group II)			
Case ID	Tumour Nodule	NSCLC Subtype	Tumour Grad	Case ID	Tumour Nodule	NSCLC Subtype	Tumour Grad
L01	TN1	SQ	G2	L09	TN1	AD	G3
	TN2		G3		TN2	SQ	G3
L02	TN1	SQ	G2	L10	TN1	AD	G3
	TN2		G2		TN2	SQ	G3
L03	TN1	AD	G2	L11	TN1	AD	G3
	TN2		G2		TN2	SQ	G3
	TN3		G2	L12	TN1	SQ	G3
	TN4		G2		TN2	AD	G3
L04	TN1	SQ	G2	L13	TN1	SQ	G3
	TN2		G2		TN2	AD	G3
L05	TN1	AD	G2 + G3	L14	TN1	SQ	G3
	TN2		G2		TN2	AD	G3
	TN3		G2 + G3	L15	TN1	SQ	G3
L06	TN1	AD	G2		TN2	AD	G3
	TN2		G2	L16	TN1	AD	G3
	TN3		G2		TN2	SQ	G3
	TN4		G2	L17	TN1	AD	G3
NSCLC of ADSQ subtype (group II)					TN2	SQ	G3
L07	TN1	AD	G3	L18	TN1	AD	G3
	TN2	SQ	G3		TN2	SQ	G3
L08	TN1	AD	G3	L19	TN1	AD	G3
	TN2	SQ	G3		TN2	SQ	G3

AD: adenocarcinoma, SQ: squamous cell carcinoma.

### DNA extraction

Lesional areas of NSCLC were marked by senior pathologists (SCS, CT, JM) and nodules with more than 80% were scraped off or -if necessary- laser microdissected as we described previously^36^. Thereafter, DNA was automatically extracted using the Maxwell DNA FFPE isolation kit on a Maxwell platform (Promega GmbH, Mannheim, GER) according to the manufacturer’s instructions. PCR accessible DNA was determined by qPCR as described previously^40, 41^. In brief, PCR amplifiable DNA was quantified by real-time PCR using the HFE gene as amplifying reference (173 bp). Standard curves in a range of 0.195 to 50 ng were prepared from unmutated high quality DNA (Takara Saint-Germain-en-Laye, F). Real-time PCR was then carried out in triplicates with 1 µl DNA each, in a 20 µl reaction mix containing 0.4 µM of the HFE forward and reverse primer (HFE-173F: TTC TCA GCT CCT GGC TCT CAT C and HFE-173R: TCG AAC CTA AAG ACG TAT TGC CC) and the GoTaq® qPCR Master Mix (Promega).

### Primer design, multiplex PCR-based library construction and next generation sequencing

To generate amplicons of a low size (around 60–200 bp), 108 primer sets spanning the whole mtDNA (Supplemental Table S1) were designed according to the mt-sequence of accession no. NC_012920 or taken from previously published primer sets (Fig. 1A, Supplemental Table S1). For enrichment of the mt-genome by a multiplex PCR, primer sets were pooled in four primer mixes of 2 µM and in each reaction, 10 ng of PCR accessible DNA -representing DNA of around 1500 cells was used (Fig. 1B).

mtDNA was then amplified in four separate multiplex PCR reactions per sample using the GeneRead DNAseq Panel PCR Kit (QIAGEN Inc., Hilden, GER) in accordance with the manufacturer´s protocol. Libraries were pooled and purified using Agencourt® AMPure® XP magnetic beads and a Biomek® FXp workstation (Beckman Coulter Inc, Fullerton, CA, USA). Fifty ng enriched targets of each sample were adenylated and ligated to NEXTflex™ DNA barcodes-48 (Bioo Scientific, Austin, TX, USA). After Agencourt® AMPure® XP magnetic bead purification and size selection, barcoded libraries were amplified by five PCR cycles. Finally, 12 pM of the constructed libraries were sequenced using the V2 chemistry of Illumina Inc. (San Diego, CA, USA) and 2 × 300 bp sequencing read length on an Illumina MiSeq platform following the manufacturer’s recommendations.

### Data filtering and analysis

The FASTQ files generated by the Illumina platform were analysed by means of the Biomedical Genomics Workbench 2.5.1 (QIAGEN Inc., Hilden, GER; www.qiagenbioinformatics.com). To determine run performance and off-target reads, the FASTQ sequences were mapped against the whole human reference genome hg19. For variant calling and annotation, the mt-genome (Genebank; accession no. NC_012920) served as a reference. Using the workflow tool of the Biomedical Genomics Workbench 2.5.1 software in batch mode ensured successive and identical analysis of all samples. The minimum read depth was set to 30, with the minimum variant frequency set to 5%. Furthermore, variant calling was restricted to loci with a balanced forward-backward performance ( > 0.2). Polymorphisms were recognised using the MITOMAP (http://www.mitomap.org/bin/view.pl/MITO-MAP/HumanMitoSeq), dbSNP-v138 (http://www.ncbi.nlm.nih.gov/SNP/_id=138) and HAPMAP_phase_3 http://hapmap.ncbi.nlm.nih.gov/hapmap3r3_B36/) databases. Spurious calls were subsequently filtered by manual analysis using the integrative genomic viewer of the Biomedical Genomics Workbench software. Variants, which occur in different sample sets but with a similar frequency as well as variants which were located in repetitive or highly homologous regions of the mt-genome, in high background noise regions, or at the end of the amplicons were considered as putative false variants. Potential false positive variants were either deleted when they were clearly recognizable as artefacts or were further re-assessed by Sanger sequencing. In addition, whenever DNA was still available, the mt-DNA regions carrying a variant in one lesion sample but not in another of the same patient sample set, were subsequently re-analysed by conventional Sanger sequencing.

### Data Availibility

All data generated or analysed during this study are included in this published article and its Supplementary Information files. Filtered variant profiles of wmt-seq can be found in supplemental Table 4, showing variants of the sample sets in the different excel sheets. Raw data are available as FASTQ sequences at the SRA data base (SUB2583044 and SUB2962918).

## Electronic supplementary material

Supplemental Figures S1-S4

Supplemental Table S1

Supplemental-Table S2

Supplemental-Table S3

Supplemental Table S4
