# Supplementary material for: Evolution analysis of heterogeneous non-small cell lung carcinoma by ultra-deep sequencing of the mitochondrial genome
Source: Sci Rep. 2017 Sep 11;7:11069. doi: 10.1038/s41598-017-11345-3 (PMC5593826; doi:10.1038/s41598-017-11345-3)
Supplement: Supplementary file 1 — Supplemental Figures S1-S4 [file 41598_2017_11345_MOESM1_ESM.doc]

**Evolution analysis of heterogeneous non-small cell lung carcinoma by ultra-deep sequencing of the mitochondrial genome**

**Running Title: Tumour tracking by ultra-deep sequencing of the entire mitochondrial genome**

Wafa Amer^1^, Csaba Toth^1^, Erik Vassella^2^, Jeannine Meinrath^1^, Ulrike Koitzsch^1^, Anne Arens^3^, Jia Huang^1^, Hannah Eischeid^1^, Alexander Adam^1^, Reinhard Buettner^1,4,5,6^, Andreas Scheel^1,4,5^, Stephan C. Schaefer^1,4,5#^, and Margarete Odenthal^1,4,6 ,7#^

**SUPPLEMENTARY MATERIAL**

**Supplemental Figure S1**

**Supplemental Figure S2**

**Supplemental Figure S3**

**Supplemental Figure S4**


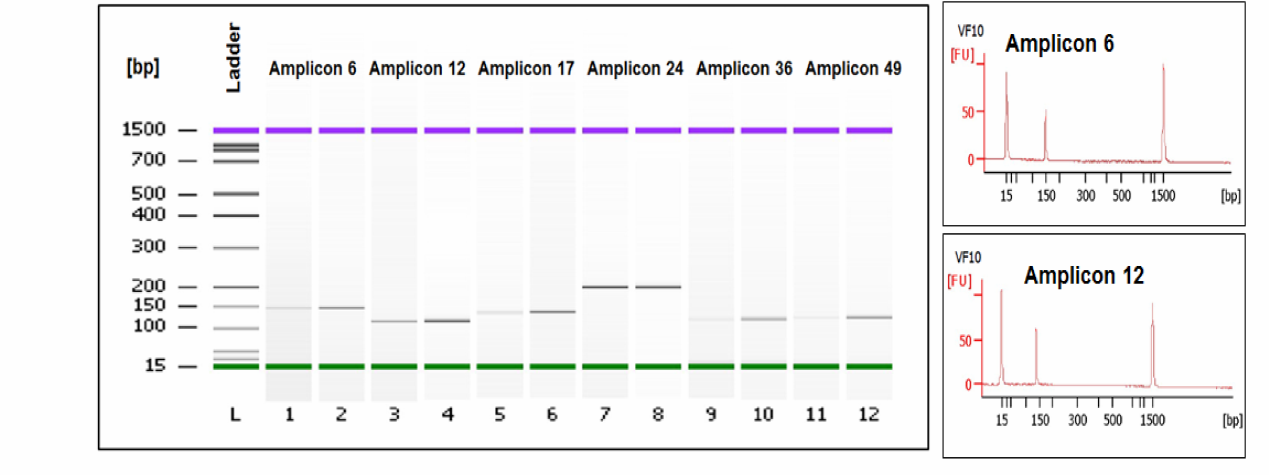


**Figure S1**:

**Efficient PCR amplification of mt-regions by designed mt-primers.** The PCR products resolved by microfluidic-based electrophoresis showed a single sharp bands in the range of the molecular weight, estimated from the respective primer sets.


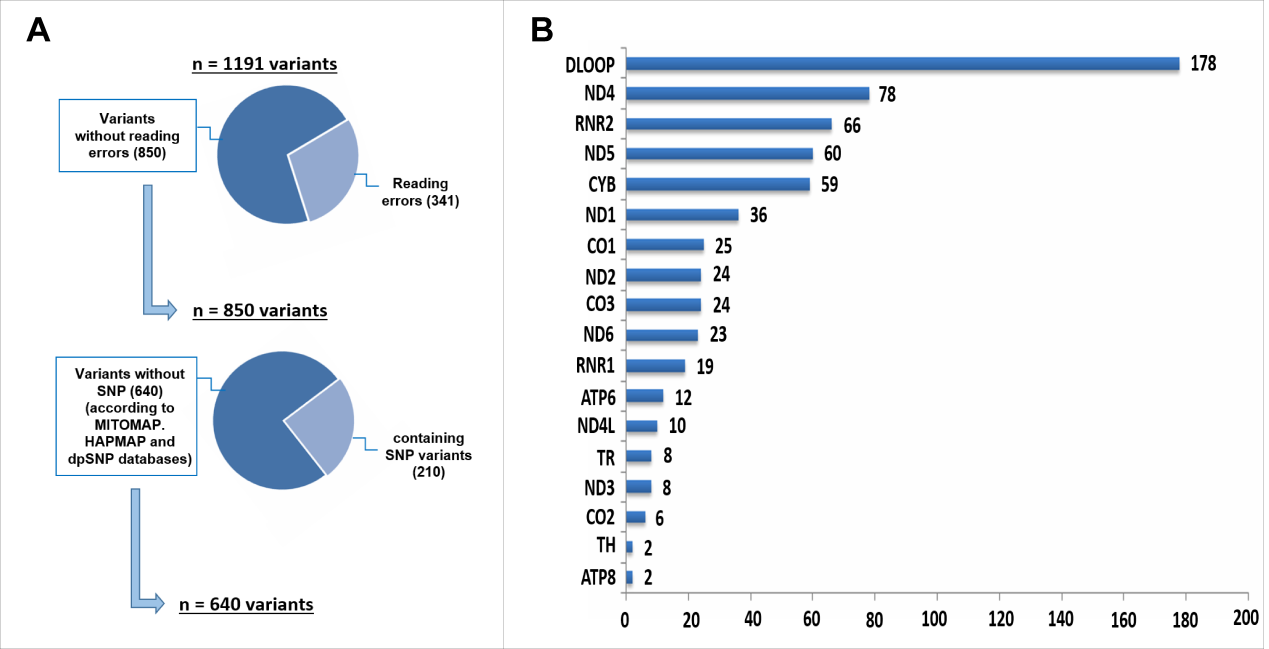


**Figure S2**:

**Workflow of NGS data interpretation and mt-variant frequencies.** After deletion of the reading errors and dbSNP variants as described in MITOMAP (<http://www.mitomap.org/bin/view.pl/MITOMAP/HumanMitoSeq>), dbSNP-v138 (<http://www.ncbi.nlm.nih.gov/SNP/snp_summary.cgi?view+summary=view+summary&build_id=138>) and HAPMAP_phase_3 http://hapmap.ncbi.nlm.nih. gov/cgi-perl/gbrowse/hapmap3r3_B36/) databases from total sequencing variant output, 640 reliable variants remain (A). The most frequent mt-variants are located at the D-LOOP regulatory site for replication and expression, and in genes of respiratory chain complex particularly in the ND4 gene (B).


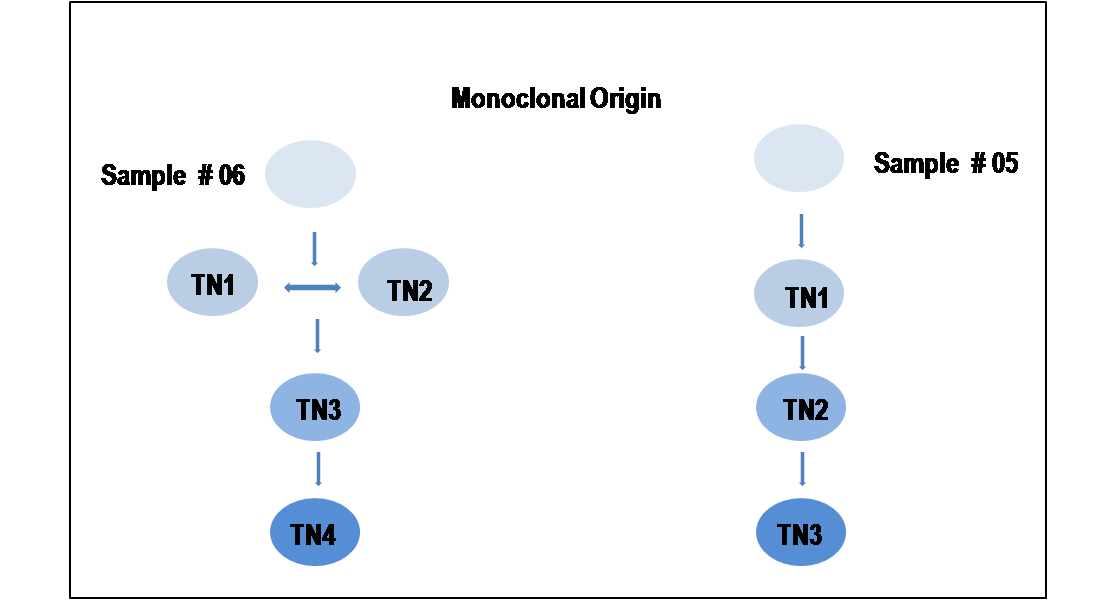


**Figure S3**: **Tumor evolution of NSCLC case #06 and case #05**

Somatic branch mt-mutations indicate the NSCLC history of tumour nodules TN1 to TN4 of case #06 and of nodules TN1 to TN3 of case #05. In particular, increasing frequencies of branch mt- variants of NSCLC #5 (Supplemental Table 4) show a linear expansion of the tumor nodules.


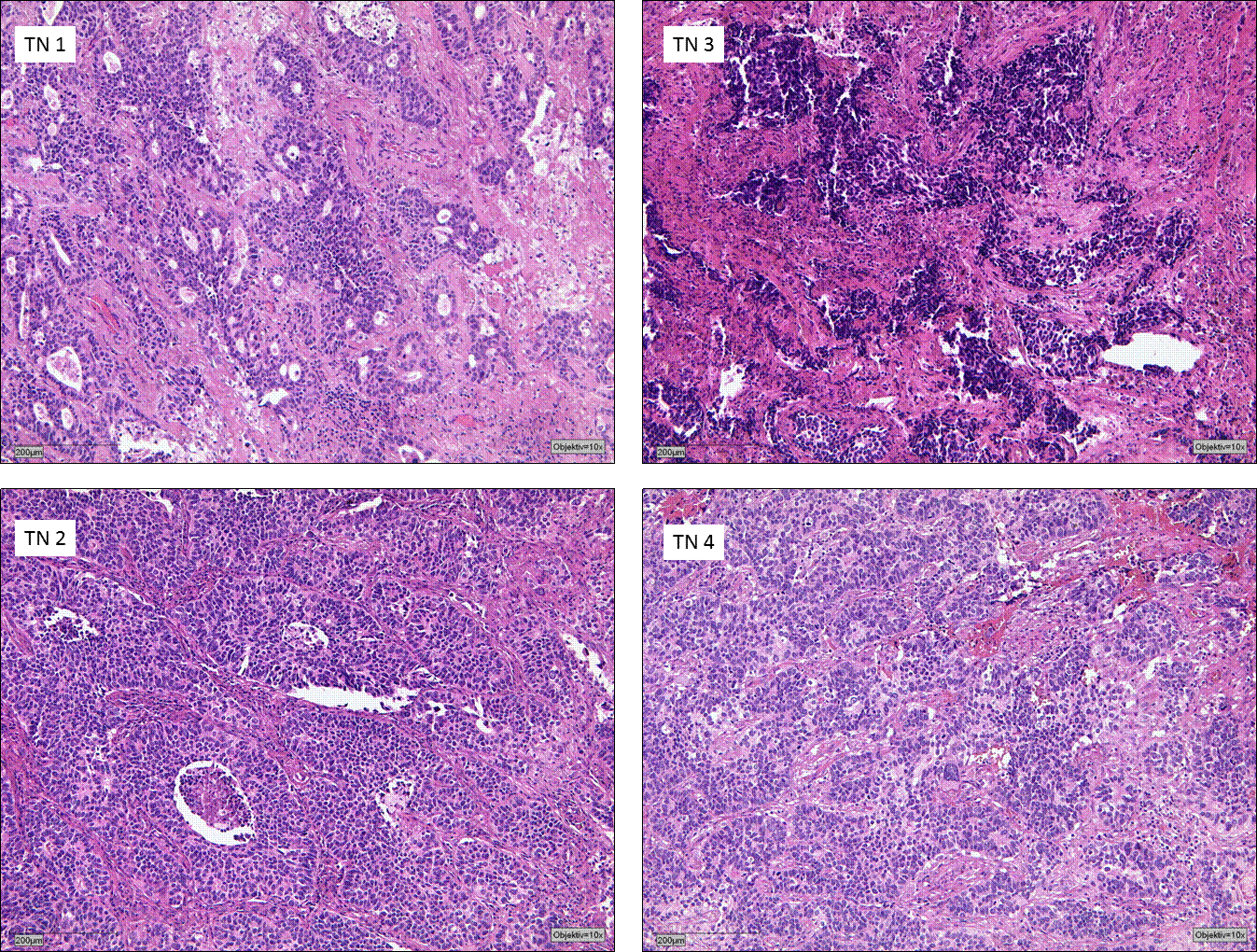


**Supplemental Figure S4:**

Histologically different aspects of NSCLC case (#03) within four separate tumor nodules. Representative images of four different tumor nodules within the same lung lobe. Gland formation (TN1), comedo-type necrosis (TN2) and solid growth pattern (TN3 and TN4). Adenocarcinoma differentiation of all four nodules was confirmed by immunohistochemical staining (not shown).
